# Clinical Characteristics and Self-Harm in Forensic Psychiatric Patients

**DOI:** 10.3389/fpsyt.2021.698372

**Published:** 2021-08-02

**Authors:** Natalie Laporte, Andrejs Ozolins, Sofie Westling, Åsa Westrin, Märta Wallinius

**Affiliations:** ^1^Child and Adolescent Psychiatry, Department of Clinical Sciences Lund, Lund University, Lund, Sweden; ^2^Centre for Ethics, Law, and Mental Health, Institute of Neuroscience and Physiology, The Sahlgrenska Academy at the University of Gothenburg, Gothenburg, Sweden; ^3^Research Department, Regional Forensic Psychiatric Clinic, Växjö, Sweden; ^4^Department of Psychology, Linneaus University, Växjö, Sweden; ^5^Department of Clinical Sciences Lund, Psychiatry, Lund University, Lund, Sweden; ^6^Office of Psychiatry and Habilitation, Psychiatric Clinic Lund, Lund, Sweden; ^7^Office for Psychiatry and Habilitation, Psychiatry Research Skåne, Lund, Sweden

**Keywords:** self-harm, non-suicidal self-injury, suicide attempt, forensic psychiatric patients, psychiatric disorders, ISAS scale

## Abstract

Self-harm, comprising non-suicidal self-injury, and suicide attempts, is a serious and potentially life-threatening behavior that has been associated with poor life quality and an increased risk of suicide. In forensic populations, increased rates of self-harm have been reported, and suicide is one of the leading causes of death. Aside from associations between self-harm and mental disorders, knowledge on self-harm in forensic psychiatric populations is limited. The purpose of this study was to characterize the clinical needs of a cohort of forensic psychiatric patients, including self-harm and possible risk factors thereof. Participants (*N* = 98) were consecutively recruited from a cohort of forensic psychiatric patients in Sweden from 2016 to 2020. Data were collected through file information, self-reports, and complemented with semi-structured interviews. Results showed that self-harm was common among the participants, more than half (68.4%) of whom had at some point engaged in self-harm. The most common methods of non-suicidal self-injury were banging one's head or fist against a wall or other solid surface and cutting, and the most common method of suicide attempt was hanging. The most prominent functions of non-suicidal self-injury among the participants were intrapersonal functions such as affect regulation, self-punishment, and marking distress. Self-harm in general was associated to neurodevelopmental disorders (*p* = 0.014, CI = 1.23–8.02, OR = 3.14) and disruptive impulse-control and conduct disorders (*p* = 0.012, CI = 1.19–74.6, OR = 9.41), with reservation to very wide confidence intervals. Conclusions drawn from this study are that self-harm was highly prevalent in this sample and seems to have similar function in this group of individuals as in other studied clinical and non-clinical groups.

## Introduction

Every year 800 000 people in the world commit suicide. This corresponds to one suicide every 40 s (1). In forensic populations, i.e., offenders with or without varying degrees of mental disorders, suicide is one of the leading causes of death (2, 3), and it has been reported that suicide is five to 10 times higher in prison populations than in general populations (2, 4, 5). Studies in prison settings have found some environmental factors (e.g., being in a single cell), psychiatric factors (previous suicide attempts, recent suicide ideation, mental illness), and criminological factors (being on remand, having received a life sentence, and having a violent index offense) particularly important in identifying individuals with a high risk of suicide (6). One of the main risk factors for suicide in prison populations is previous non-suicidal self-harm behavior; the risk of completed suicide has been found to be 30 times higher among people who demonstrate non-suicidal self-harm behavior than among those who do not (6–8).

The term self-harm is broad and refers to both non-suicidal self-injury (NSSI) and self-inflicted harm with the intention of committing suicide (suicide attempt) (9). This behavior is considered a global public health issue and is common in the general population (2.9–41.5%) (10, 11). In prison settings, the prevalence of non-suicidal self-harm and suicide attempts has been reported to vary from 7 to 47.6% (12, 13). In a Swedish prison cohort, the actual lethal intention of apparent suicide attempts was found to be as low as 6% (14). To our knowledge, few studies discuss the intention of suicide attempts. However, one study found that individuals with personality disorders had significantly lower intention of completed suicide than those with substance use or unknown psychiatric disorders (15). In sum, self-harm constitutes a significant challenge not only in parts of the general population, but also in forensic settings such as prisons. However, while it is important to determine the prevalence of such a challenging behavior, understanding why some individuals injure themselves is essential for designing and implementing treatment and prevention.

One specific setting where knowledge on self-harm is scarce is within forensic psychiatry. Every year, ~350 individuals are convicted to forensic psychiatric care in Sweden. Forensic psychiatric patients (~1,800) (16) are a relatively small group compared with the significantly larger group of people imprisoned in Sweden (~5,000) each year (17). In international comparisons, it has been demonstrated a significant variation in both the number of forensic beds available, length of care and patient group characteristics [e.g., gender distribution; (18)] Nevertheless, a common denominator for all forensic psychiatric contexts is that forensic psychiatric patients require substantial effort and skill in terms of health care and intervention. These patients' clinical presentations are characterized by a complex spectrum of mental disorders and comorbid psychosocial problems, antisocial behaviors, and early adverse experiences (16, 19). The few studies on non-suicidal self-harm and suicide attempts among forensic psychiatric patients report alarmingly high rates (~61%) (2, 20, 21). The severity of self-harm varies greatly in these populations, which raises questions about the function of this behavior. To our knowledge, this has not been studied previously in forensic psychiatric patients, but theoretical and clinical studies in other populations indicate that self-harm may function as an emotion regulation strategy (22–28).

The clinical presentations and overrepresentation of self-harm in forensic psychiatric patients make clear that this population is extremely vulnerable in this area. Forensic psychiatric care urgently needs to help these patients, but knowledge upon which to base evidence-based practice is scarce.

## Aims

The explorative purpose of this study was to describe the clinical characteristics of a cohort of consecutively recruited forensic psychiatric patients with non-suicidal self-injury and suicide attempts and possible risk factors thereof, with the following specific aims:

1. Describe the psychosocial, criminological, and psychiatric characteristics of a cohort of forensic psychiatric patients,
2. Determine the prevalence, characteristics of non-suicidal self-injury and suicide attempts and functions of non-suicidal self-injury in forensic psychiatric patients,
3. Identify possible psychosocial and clinical risk factors of non-suicidal self-injury in forensic psychiatric patients.

## Methods

### Participants

This study was conducted in a consecutively recruited cohort of forensic psychiatric patients. All patients who met the initial criterion of being cared for at a high security forensic psychiatric clinic in Sweden during the data collection period of November 2016 to November 2020 were candidates for participation. To be included, patients had to have a longer predicted stay than 8 weeks at the clinic and be able to fulfill the tasks in the study without an interpreter. Also, all patients were assessed by their treating psychiatrist prior to participation and were excluded if assessed as unable to provide informed consent. The sample included only patients sentenced to forensic psychiatric care. Patients with remand statues or ongoing prison sentences with temporary need for involuntary psychiatric care were excluded from the study.

**Figure 1:**
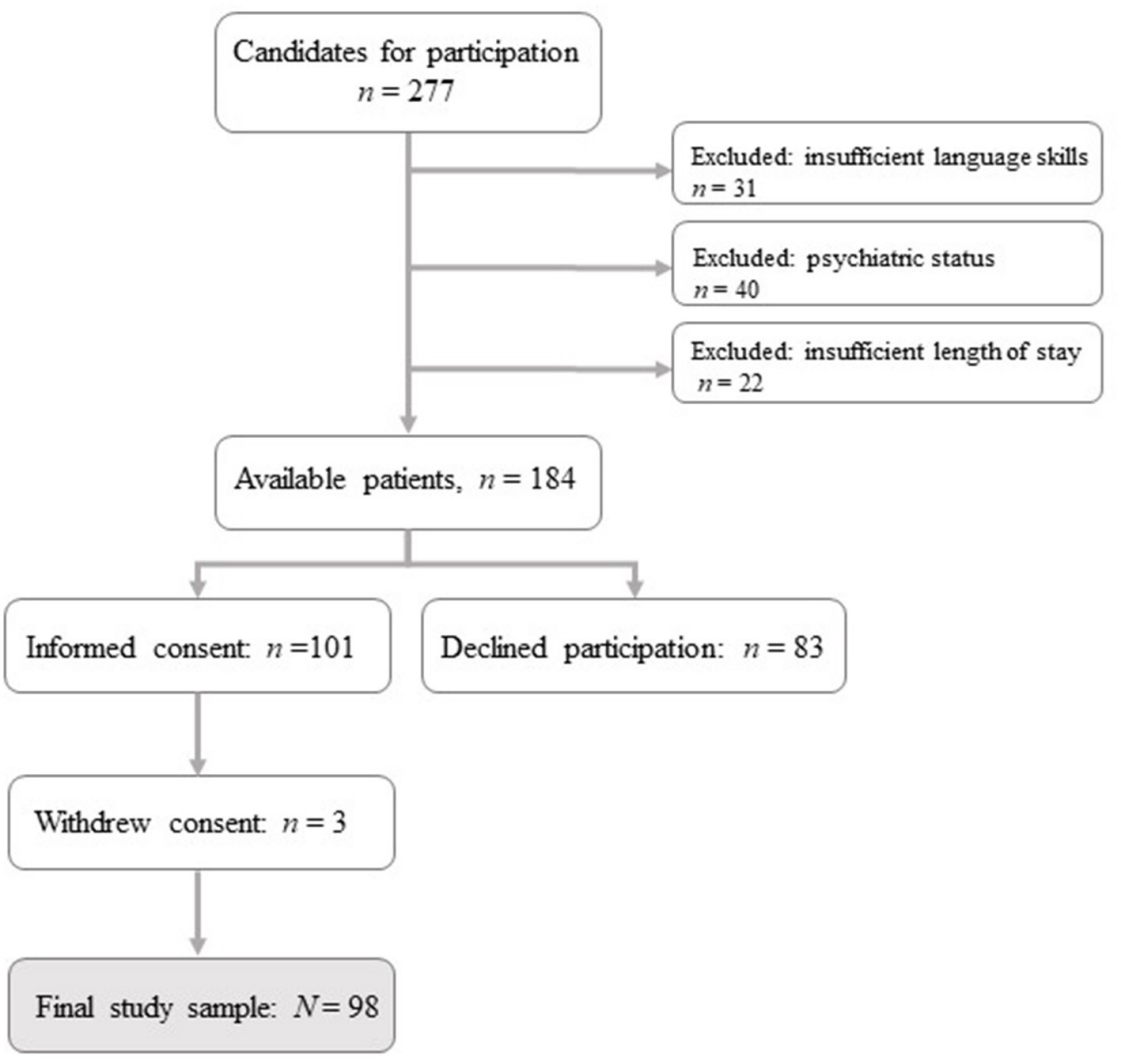
Flowchart for inclusion of participants.

The aim was to collect 100 participants, but due to the COVID-19 pandemic, inclusion of participants was terminated in November 2020 after 98 patients had participated. The study was based on 98 participants (56% participation rate). For a detailed overview of the inclusion of participants (see Figure 1).

The mean age of the participants was 34.9 years (range 19–62, *SD* = 10.7) and 86.7% were male (*n* = 85). The mean length of stay in the current forensic psychiatric care period was 23.5 months (range 1–135, *SD* = 33.5), with most participants (*n* = 87, 88.8%) being treated under special care supervision, indicating a significant risk of recidivism. Only 14.3% (*n* = 14) of the participants had previously been in forensic psychiatric care. According to the Swedish National Forensic Registry report from 2019, the median age of forensic psychiatric patients in Sweden was 40 years, and 84% of the patients were male. The majority (90% for males and 84% for females) were being treated under special care supervision and 14% of the male patients and 11% of the female patients had previously been under forensic psychiatric care. Given this, the current sample seems representative of the population forensic psychiatric patients in Sweden. However, during data collection, nine participants chose to terminate their participation before all data had been collected and one self-report was assessed as unreliable. The characteristics of the nine patients who chose to terminate their participation could be summarized by the following: 90% male, all with different current primary diagnoses and index crimes. Since the participants had been informed that they could terminate their participation at any time without giving a cause, no data on reason of dropout is available.

### Procedures

Information on the study was given to all 184 eligible participants by one of the two data collectors (the first author and a fellow PhD student), both with clinical experience with forensic psychiatric patients. After receiving oral and written information on the study, those who chose to participate provided written informed consent. Thereafter, the data collectors gathered all available file information, including the forensic psychiatric investigation (FPI), medical records from psychiatric health care facilities, detailed reports on previous living circumstances and criminal history, written court verdicts, and incidents during current treatment. The data collectors then met each participant, on one or several occasions depending on the participant's needs, to conduct self-report questionnaires. When the information from files was considered insufficient, complementary semi-structured interviews were conducted. A data collector was present for all participants while they answered the questionnaires to provide any necessary support (e.g., emotional support or interpretation of questions). After data collection was completed for each participant, all data were assessed for quality through a review by the data collector and a senior clinician and researcher in the field. Every participant received a small monetary compensation for their contribution to the study.

### Measures

#### Psychosocial Background

Sociodemographic information (e.g., age and gender) and information on psychosocial background (e.g., schooling, institutionalization during childhood, work experience, alcohol and substance use), and information on previous psychiatric health care was obtained from files and complemented with interviews with the participant. Information on psychosocial background (e.g., parents absent during childhood) was asked as “Did the participant grow up with one or both parents absent during a significant part of their childhood?” and responses were categorized as “No,” “Yes, mother absent,” “Yes, father absent,” or “Yes, both parents absent.” Information on institutionalization was divided into two categories: shorter stay (<4 weeks) and longer stay (≥4 weeks). Information on previous criminality was collected through the FPI and retrieving written court verdicts from the local district court. Criminal behaviors were categorized as follows: lethal violence (murder/manslaughter), assaults (not lethal or sexual), other violent crimes (threats and violence against an officer, unlawful threat, and fire setting/arson), sexual assaults (all sexual acts prohibited by the Swedish Penal Code), theft or robbery, economic crimes, traffic offenses, drug offenses, and unlawful possession of weapons. Responses were then divided into: “No,” “Yes, single occasion (one time),” or “Yes, repeated occasions (two times or more).”

#### Mental Health

Clinical factors regarding mental health including substance use disorders, both lifetime occurrence and current primary and secondary diagnoses of mental disorders, were collected through medical files and the FPI. In the files, diagnoses were specified in DSM-IV (29), ICD-9 (30), or ICD-10 (31) format and were therefore converted to DSM-5 (32) by a senior clinician, a psychologist and researcher (author MW) with considerable experience in the field. Information on diagnoses was categorized into (1) current diagnoses (primary and secondary) and (2) diagnoses at any point in a participant's life (from child and adolescent psychiatry until current stay within forensic psychiatric care). We found that one participant had a schizophrenia diagnosis both as a current main diagnosis and as a secondary diagnosis. This proved to be a miscoding in the medical file, and the patient was coded in our study as having schizophrenia only as primary diagnosis.

#### Self-Harm

Information on lifetime self-harm was collected from files and self-reports, complemented by interviews. Data on NSSI (any occasion, number of occasions, age at onset, type of self-injury, and function of the behavior) and suicide attempts (any attempt, age at onset, violent attempts, risk of completed suicide at most serious attempt) were collected separately. The self-report instrument Inventory of Statements About Self-injury (ISAS) (33), designed to comprehensively assess the frequency and functions of NSSI, was also used to collect information on NSSI. The ISAS assesses NSSI in two parts: (1) the lifetime frequency of 12 NSSI made intentionally but without suicidal intent, and (2) the 13 functions of NSSI. In the first part of the ISAS, participants are asked to estimate the number of times they have used specific methods of NSSI. Additional multiple-choice questions assess descriptive and contextual factors including age at onset, pain experienced during the NSSI act, whether the behavior is performed alone or in the presence of others, time between the first urge to self-harm and the actual act (<1, 1–3, 3–6, 6–12, 12–24 hr, and >1 day), and whether the participant wants to stop self-harming. Only participants who confirmed one or more NSSI behaviors in the first part were asked to proceed to the second. The second part evaluates the 13 potential functions of NSSI by three items per function rated as “0: not relevant,” “1: somewhat relevant,” or “2: very relevant”: affect regulation, anti-dissociation, anti-suicide, autonomy, interpersonal boundaries, interpersonal influence, marking distress, peer bonding, self-care, self-punishment, revenge, sensation seeking, and toughness. Scores for each function range from 0 to 6. These 13 functions constitute two overall factors: interpersonal factors (e.g., interpersonal influence, peer bonding), and intrapersonal functions (affect regulation, self-punishment) (33). The ISAS factors have previously presented good internal consistency and expected correlations with both clinical and contextual factors, supporting the reliability and validity of ISAS (34). The Swedish translation of the ISAS has demonstrated good internal consistency for the interpersonal and intrapersonal factors in a female population with known and severe self-harm (35). The ISAS has not been validated in forensic settings with an explorative objective, nor has any other self-report assessment of self-harm. In this study, Cronbach's alpha was used to calculate internal consistency for the ISAS self-report items, demonstrating good internal consistency (α = 0.898 for the intrapersonal scale and α = 0.859 for the interpersonal scale; both over the acceptable value of 0.7). Analyses on ISAS-factors were performed on the 43 participants who had answered the ISAS.

In the Results section we specify suicide attempts because we believe this is of clinical relevance. Participants were asked “Have you ever made a suicide attempt with the intention to die?” Participants who answered “Yes” were asked to report their most recent method of suicide attempt, any attempt of suicide in the last 6 months, any substance use in conjunction with the attempt, and the lethality of the latest attempt. Levels of lethality of the attempt were categorized using the scale from C-SSRS Suicide risk assessment instrument, “Actual Lethality/Medical Damage,” categorizing the physical consequences of suicide attempts on a 6-point Likert scale (0–5) (36).

### Statistical Methods

For the first and second aim, we used descriptive frequency tables to report psychosocial, criminological, and clinical backgrounds and information on self-harm. For the third aim, we performed chi-square tests of independence to examine associations between self-harm and psychosocial and clinical factors deemed relevant based on previous research. We performed all bivariate analyses with the general self-harm variable as dependent variable, which was created by merging two variables (suicide attempt yes/no and NSSI yes/no). Effect sizes, confidence intervals, and odds ratios (ORs) were reported for ease of interpretation. Several diagnoses could not be analyzed in relation to self-harm due to a low number of participants in each cell (see **Table 5** in the Results section for more information). The authors are aware of the large variation of mental disorders in this population, and that a small representation in some disorder categories might lead to statistical power issues. However, this is an explorative study why we argue for the need to examine the sample thoroughly regarding this issue. We did not correct for multiple comparisons because of the explorative purpose of the study.

### Ethical Considerations

Because of the studied population's vulnerabilities, ethical considerations were especially important. We consulted the treating forensic psychiatrist for all candidates for participation and excluded all candidates considered not currently suitable for the study due to psychiatric status (e.g., acute psychosis or imminent risk of violence) or unable to provide informed consent (e.g., due to intellectual disability). All participants provided voluntary informed written consent before participation and were informed of their right to terminate participation at any time without giving a reason. The study, including the small monetary reward (low in order not to give an incentive that would compromise free consent), was approved by the Research Ethics Committee at Linköping University, 2016/213-31 and 2017/252-32.

## Results

### Psychosocial, Criminological, and Clinical Characteristics of Forensic Psychiatric Patients

The psychosocial backgrounds of the participants are presented in Table 1. For gender-specific distributions, see Table A1. A subgroup of the participants had not graduated from compulsory primary school (*n* = 19, 19.6%), while 25 participants (25.8%) had completed high school. A minority had initiated studies at the university level (*n* = 6, 6.2%) or completed a vocational training education (*n* = 4, 4.1%). As reported in Table 1, almost one in three of the participants had bullied other children during childhood, with the majority (*n* = 21, 22.6% of the total cohort) having done so repeatedly. Truancy was reported for more than three in four of the participants, with many (*n* = 58, 61% of the total cohort) demonstrating a high rate of truancy. Among the participants who grew up with one or both parents absent, 27 (27.6%) reported one single parent as absent, while in 13 cases (13.3%) both parents had been absent during a significant time of their childhood. About one in three participants had been institutionalized during childhood, and longer stays (several months or years) was more frequent (*n* = 32, 32.7%) than shorter stays (a couple of weeks; *n* = 4, 4.1%). This was also the case with foster care placements, where a longer stay was more frequent (*n* = 21, 21.4%) than a shorter stay (*n* = 7, 7.1%). The gender-specific distributions presented in Table A1 in the Appendix, demonstrated some trends regarding gender differences, e.g.,; female participants were more often than males in some kind of a partner relationship, reported much less work experience than their male counterparts, and had to a lower degree bullied others during childhood.

**Table 1:** Psychosocial background of forensic psychiatric patients (*N* = 98).

**Background characteristic**	***n***	**%**
**Born in Sweden**	70	71.4
**Marital status**
Single	84	86.6
In a partner relationship/married	13	13.4
Parent of a child	27	27.6
**Schooling**
Graduated from primary school	43	44.3
Truancy	74	77.9
Bullied others	29	31.2
**Work experience**
Full-time employment for >1 year	33	34
Part-time employment for >1 year	19	19.6
**Upbringing circumstances**
Parent(s) absent during childhood	40	40.9
Institutionalization before age 18	36	36.8
Foster care placement	28	28.5

The mean age at first prosecuted offense was 22.3 years (median = 18, range 15–50) among the participants, and the mean age of onset at first crime (not prosecuted) was 14.7 (median = 14, range 6–47). For male participants, the age range of first prosecuted offense was 15–50, while for female participants the range was 20–41 (see Table A2). The number of previous convictions per participant ranged from 1 to 50, with a mean number of convictions at 7.4 for the whole cohort. The maximum number of previous convictions reported among the female participants was 6 times. The mean number of prison sentences was 1.7 (range 0–38). Female participants who had committed an offense of lethal violence (*n* = 4, 30.8%) had done so at a single occasion. No woman had committed an offense of lethal violence at multiple occasions. Overall, the majority of the female participants reported assaults (*n* = 11, 84.6%), other violent crimes (non-sexual) (*n* = 11, 84.6%), theft or robbery (*n* = 10, 77%), and drug offenses (*n* = 10, 77%). For detailed information on the criminological background of the cohort, see Table 2 and Table A2 for gender-specific distributions.

**Table 2:** Criminological characteristics of forensic psychiatric patients.

**Type of offense**	**Yes** **single occasion** ***n* (%)**	**Yes** **repeated occasions *n* (%)**	**No** ***n* (%)**	**Age at onset,** **mean (range)**
Lethal violence	20 (20.4)	5 (5.1)	73 (74.5)	27.6 (19–41)
Assaults (non-sexual)	22 (22.4)	59 (60.2)	17 (17.3)	19.3 (5–47)
Other violent crimes (non-sexual)	13 (13.3)	76 (77.6)	9 (9.2)	23.1 (7–50)
Sex offences	6 (6.1)	6 (6.1)	83 (87.4)	22.4 (11–39)
Theft or robbery	19 (19.4)	70 (71.4)	9 (9.2)	16.4 (5–45)
Economic offenses	11 (11.3)	15 (15.5)	71 (73.2)	22.2 (13–36)
Traffic offenses	27 (27.8)	40 (41.2)	30 (30.9)	20.2 (11–35)
Drug offenses	4 (4.1)	76 (77.6)	18 (18.4)	15.9 (8–35)
Unlawful weapons possession	22 (22.4)	37 (37.8)	39 (39.8)	21.1 (9–47)

As seen in Table 3, a majority of the participants had a current or history of diagnosis within the spectrum of schizophrenia or other psychotic disorders. The most frequent current primary diagnosis at time of participation in this spectrum was schizophrenia (*n* = 19, 19.4%), predominantly paranoid or unspecified, followed by unspecified schizophrenia or other psychotic disorder (*n* = 18, 18.4%). A common category in previous diagnoses was substance-related and addictive disorders, with almost two in three (62.2%) participants having received such a diagnosis at some point during their lifetime. The most common substance use disorder was “Other” or “Unknown)” (*n* = 37, 37.8%), followed by cannabis-related disorders (*n* = 20, 20.4%) and stimulant-related disorders (*n* = 15, 15.3%).

**Table 3:** Current and historical mental disorders in forensic psychiatric patients.

**Diagnosis**	Lifetime prevalent diagnosis^*^ ***n* (%)**	Current primary diagnosis^*^ ***n* (%)**	Current secondary diagnosis^*^ ***n* (%)**
**Neurodevelopmental disorders**	46 (46.9)	21 (21.4)	23 (23.5)
Intellectual disability, any kind	13 (13.3)	0	0
Attention-deficit/hyperactivity disorder	34 (34.7)	4 (4.1)	16 (16.3)
Autism spectrum disorder	25 (25.5)	14 (14.3)	7 (7.1)
**Schizophrenia spectrum and other psychotic disorders**	69 (70.4)	51 (52.0)	7 (7.1)
**Bipolar and related disorders**	11 (11.2)	5 (5.1)	2 (2.0)
**Depressive disorders**	24 (24.5)	1 (1.0)	0
**Anxiety disorders**	28 (28.6)	0	0
**Obsessive-compulsive and related disorders**	7 (7.1)	0	1 (1.0)
**Trauma- and stressor-related disorders**	18 (18.4)	0	4 (4.0)
Post-traumatic stress disorder	8 (8.2)	0	3 (3.1)
Other trauma and stressor-related disorders	13 (13.3)	0	2 (2.0)
**Disruptive, impulse-control, and conduct disorders**	17 (17.3)	1 (1.0)	5 (5.1)
Oppositional defiant disorder	5 (5.1)	0	1 (1)
Intermittent explosive disorder	5 (5.1)	0	1 (1)
Conduct disorder	5 (5.1)	0	0
Unspecified disruptive, impulse-control, and conduct disorder	7 (7.1)	0	3 (3.1)
**Substance-related and addictive disorders**	63 (64.3)	2 (2)	32 (32.7)
**Personality disorders, any**	42 (42.9)	18 (18.4)	12 (12.2)
Cluster A personality disorders	7 (7.1)	0 (0)	0 (0)
Cluster B personality disorders	38 (38.8)	12 (12.2)	18 (18.4)
Cluster C personality disorders	1 (1.0)	0 (0)	0 (0)
Other personality disorders	25 (25.5)	4 (4.1)	5 (5.1)
**Paraphilic disorders**	2 (2.0)	1 (1.0)	1 (1.0)
**Other mental disorders**	10 (10.2)	2 (2.0)	0

*Lifetime prevalent diagnoses = diagnoses from childhood until current forensic psychiatric care; Current primary and secondary diagnoses = diagnoses at time of participation.

Two out of five participants had a history of a childhood-onset mental disorder that continued, as a primary or secondary diagnosis, at the time of participation (see Table 3). Over a lifetime perspective, attention deficit/hyperactivity disorder was the most common neurodevelopmental diagnosis among the participants.

Personality disorders were common among the participants, with two in five having a history of such a diagnosis and one in three having a current primary or secondary diagnosis (see Table 3). The most common were cluster B personality disorders, with a prevalence of antisocial personality disorder (APD) at 23.5%, *n* = 23, (*n* = 22, 25.9% of male participants, and *n* = 1, 7.7% of female participants) and borderline personality disorder (BPD) at 20.4%, *n* = 20 (*n* = 9, 10.6% of male participants, and *n* = 11, 84.6% of female participants). However, the prevalence of APD or BPD as a current primary or secondary diagnosis was low (APD primary: *n* = 7, 7.1%; APD secondary: *n* = 11, 11.2%; BPD primary: *n* = 4, 4.1%; BPD secondary; *n* = 7, 7.1%). Specific personality disorders in the other clusters were uncommon and ranged from 0 to 3 in lifetime occurrence and 0 to 1 in current diagnoses. As seen in Table A3, in the Appendix, gender differences in psychiatric (co-)morbidity were visible, except for substance-related and addictive disorders and specific disruptive, impulse-control, and conduct disorders. This was valid for both lifetime prevalence and current diagnoses.

Comorbidity was common in this sample at the time of participation. The majority of the participants had one secondary diagnosis (*n* = 43, 43.9%), 15 (15.3%) had two additional diagnoses, 10 (10.2%) had three, and 3 (3.1%) participants had four additional diagnoses. The most common secondary diagnoses were substance-related and addictive disorders. Some diagnoses belonging to the spectrum of disruptive, impulse-control, and conduct disorders had a low lifetime occurrence or no representation in this sample (Pyromania: *n* = 0 [0%], Kleptomania: *n* = 1 [1%], and Other specified disruptive, impulse-control, and conduct disorder: *n* = 3 [3.1%]).

### Prevalence, Characteristics, and Function of Self-Harm in Forensic Psychiatric Patients

In total, 67 (68.4%) of the participants had engaged in self-harm (non-suicidal self-injury and/or suicide attempts) at some point during their lifetime. Of those, *n* = 54 (55.1%) were male. All female participants in the study (*n* = 13) reported a history of NSSI or suicide attempt. Fifty-seven (58.2%) of the participants had made one or more suicide attempts, seven (12.5%) during the previous six months. Only one (*n* = 1) of the female participants had never attempted suicide. The mean age at first suicide attempt was 21.5 years of age (median 19 years; range 9–53, *SD* = 9.0). Most recent suicide attempts included several different methods. Of alternatives listed, hanging was the most common (*n* = 14, 26.4%), followed by self-poisoning (*n* = 12, 22.6%), cutting (*n* = 9, 17%), self-strangulation (*n* = 5, 9.4%), choking/swallowing objects (*n* = 3, 5.7%), jumping from heights (*n* = 2, 3.8%), and traffic related attempts (*n* = 2, 3.8%). Six (11.3%) participants had made another type of suicide attempt not given as an alternative. Asked to specify their method, they reported “caused infection,” “ran out on an iced lake,” “drove a car into a tree,” “started a fire in prison cell,” “injected air into blood,” and “tried to overdose.” The physical consequences of the participants' most serious suicide attempts were none or minimal for 20 (40%), minor for 8 (16%), moderate for 11 (22%), moderately difficult for 6 (12%) and severe or nearly lethal for 5 (10%). The most commonly used method of suicide attempt for male and female participants, respectively were hanging/strangulation for men (*n* = 13, 15.3% of male participants), and cutting for women (*n* = 6, 46.2% of female participants).

More than half of the participants (*n* = 56, 59%) had engaged in NSSI (mean age at onset 18 years, *SD* = 8.3, range 4–41). The mean age at the last episode was 28.25 years (*SD* = 8.3, range 13–45). The majority of those who had self-harmed with non-suicidal intent had not done so under the influence of drugs (*n* = 31, 66%) and, although, other data on the exact circumstances of the NSSI episode were not collected, many participants often told the data collector that these episodes had occurred during their arrest or early in their admission to forensic psychiatry. The most common method of NSSI was banging or hitting oneself (*M* = 31 occasions) along with cutting (*M* = 30 occasions). The majority of the participants who reported cutting as an NSSI (*n* = 13, 14.9%) had only done so once. Male participants reported more single occasions of cutting, while female participants reported mostly repeated occasions of cutting. The lowest frequency of cutting reported by female participants was 10 times (*n* = 3), and the rest of the female participants (*n* = 7) who had cut themselves reported high frequencies (50–1,000 times). Several participants who scored high on frequencies of NSSI stated that the frequency was impossible to count and therefore reported an estimation. Regarding pain experience while self-harming, almost half of the participants who had self-harmed (*n* = 21, 45.7%) stated “yes,” 14 (30.4%) stated “sometimes,” and 11 (24%) stated “no.” The majority (*n* = 38, 82.6%) reported that they preferred being alone while self-harming. The participants were also asked to estimate a time interval from their first thought of self-harm to the self-harm act. The majority (*n* = 31, 70%) reported “ <1 h,” 11% (*n* = 5) answered “1–3 h,” 9% (*n* = 4) answered “3–6 h,” and 6.6% (*n* = 3) answered “6–12 h” or “more than 1 day”. When asked if they wanted to stop self-harming, 81.8% (*n* = 36) of the participants answered “yes.”

Overall, the participants reported intrapersonal functions as the more relevant functions of NSSI. As seen in Table 4, the two most commonly reported functions of NSSI were affect regulation and self-punishment, followed by distress signaling. The distribution of the participants' self-reported NSSI functions were, for the majority of the scales, positively skewed, explaining the large SD for some of the scales in Table 4. See Table A4 for gender-specific distributions.

**Table 4:** Functions of NSSI (mean ISAS values) in forensic psychiatric patients.

**ISAS scale**	**Function**	***M (SD)***	**Range**
**Intrapersonal**			
	Affect regulation	3.04 (2.02)	0–6
	Anti-dissociation	1.55 (1.80)	0–6
	Anti-suicide	1.48 (2.02)	0–6
	Marking distress	2.23 (1.84)	0–6
	Self-punishment	2.48 (1.84)	0–6
**Interpersonal**			
	Autonomy	0.40 (1.07)	0–5
	Interpersonal boundaries	0.86 (1.35)	0–4
	Interpersonal influence	1.50 (1.53)	0–5
	Peer bonding	0.21 (0.51)	0–2
	Revenge	0.44 (0.88)	0–4
	Self-care	1.97 (2.07)	0–6
	Sensation seeking	0.60 (1.25)	0–6
	Toughness	0.90 (1.21)	0–4

### Psychosocial and Clinical Risk Factors of Self-Harm in Forensic Psychiatric Patients

Table 5 shows the effects of different psychosocial and clinical characteristics on self-harm when tested in chi-square analysis, demonstrating a few significant associations with wide confidence intervals. Similar results were demonstrated when analyzing only male participants (see Table A5).

**Table 5:** Psychosocial and clinical risk factors of self-harm (NSSI & suicide attempts).

Psychosocial and clinical characteristics^*^	**Self-Harm** **(***n***)**			***X^**2**^***	***P***	**CI**	**OR**
	**No**	**Yes**	**Expected yes-count**				
Female gender	0	13	8.9	6.95	0.008	1.10–1.40	1.20
Neurodevelopmental disorders	8	38	31.4	8.13	0.004	1.47–9.63	3.77
Schizophrenia spectrum and other psychotic disorders	24	46	47.9	0.79	0.372	0.24–1.72	0.64
Depressive disorders	5	19	16.4	1.71	0.190	0.69–6.15	2.06
Anxiety disorders	5	23	19.1	3.44	0.064	0.92–8.02	2.72
Trauma- and stressor-related disorders	3	15	12.3	2.28	0.131	0.72–10.09	2.69
Disruptive, impulse-control, and conduct disorders	1	16	11.6	6.31	0.012	1.19–74.58	9.41
Substance-related and addictive disorders	21	42	43.1	0.23	0.627	0.32–1.97	0.80
Personality disorder clusters A, B, and C	13	29	28.7	0.01	0.900	0.45–2.50	.90
Cluster B personality disorders	11	27	26	0.20	0.649	0.51–2.79	1.23
Other personality disorders	8	17	17.1	0.002	0.963	0.37–2.59	0.98
Parents absent during childhood	11	29	27.3	0.53	0.465	0.57–3.35	1.39
Institutionalization during adolescence	6	30	24.6	5.89	0.015	1.23–9.30	3.38
Foster care placement during childhood	6	22	19.1	1.89	0.17	0.73–5.69	2.04
Truancy	21	53	50.6	1.48	0.224	0.69–4.69	1.80
Bullying others	11	18	19.8	0.75	0.385	0.27–1.66	0.67

* *Due to low representation in some diagnostic categories, bipolar syndrome, obsessive-compulsive and related disorders, paraphilic disorders, and other mental disorders and mental illness could not be analyzed in a chi-square analysis*.

## Discussion

This study aimed to describe the clinical characteristics of self-harm and its functions and possible risk factors in a cohort of consecutively recruited forensic psychiatric patients. The participants reported many aggravating circumstances during their childhood, along with repeated criminal behaviors, both violent and non-violent, and a high prevalence and comorbidity of mental disorders, primarily within the schizophrenia spectrum and other psychotic disorders and substance-related and addictive disorders. More than half (68.4%) of the participants had at some point during their lifetime engaged in self-harm (NSSI and/or suicide attempt), and 58.2% had a history of one or multiple suicide attempts. The most commonly reported functions of NSSI were intrapersonal functions such as affect regulation, self-punishment, and marking distress, and self-harm in general was associated with neurodevelopmental disorders and disruptive impulse-control and conduct disorders, although, we acknowledge the wide confidence intervals and made no corrections for multiple comparisons. Gender differences in psychosocial, criminological and clinical characteristics were obvious, with female gender being a risk factor for self-harm.

### Psychosocial, Criminological, and Clinical Characteristics of Forensic Psychiatric Patients

Results in this study confirm previous findings that forensic psychiatric patients constitute a vulnerable group who have experienced stressful events since childhood. Many participants' childhood had been marked by seemingly complex relationships with peers and family and troubled educational histories with repeated truancy (61%) and school failures; one in five had not graduated from compulsory primary school. Almost two in five grew up without both parents present during a significant part of their childhood. Childhood institutionalization or placement in foster care, usually for long periods, was also common. Over the years, researchers have discussed the negative relationship between some children's temperaments and their parents' poor parenting and the subsequent effect on the child's behavioral adjustment in adolescent and adulthood (37, 38). Some children who are naturally more aggressive, easily frustrated, and have a hard time expressing themselves in a prosocial manner may frustrate their parents, who in response may disengage from parenting or become more sporadic and inconsistent toward the child, unfortunately intensifying the destructive development of these already vulnerable children or adolescents. The participants in our study were all once children, many of whom, for some reason, had difficulties getting through their basic education, had a high rate of truancy, and bullied their peers. For some, their childhood circumstances led to institutionalization or foster care placement for long periods of time. Taken together, the findings suggest a profound lack of parental support, something that needs to be investigated in future research. Regarding gender differences, the results suggest more externalizing childhood behaviors in male forensic psychiatric patients (e.g., bullying others), and more intimate partner relationships and lower degrees of work experience in the female patients. These are findings that are important for the rehabilitation to society for forensic psychiatric patients, since male and female patients may have different needs. Yet, this needs to be further investigated with a sample including more female patients.

The criminal histories of participants in this study included repeated assaults, threats, arson, theft, sexual violation, property crime, and drug-related crime, in accordance with reports from the Swedish National Registry of Forensic Psychiatry (16). Considering possible gender differences, the male patients demonstrated a more diverse criminological background than the female patients, with a lower age at onset confirming the suggestion above of more externalizing childhood behaviors in male forensic psychiatric patients. Male participants were overrepresented in multiple occasions of lethal violence compared to the female participants, yet the proportion of female participants that committed lethal violence at one occasion (30.8%) was larger than the proportion of male participants (18.8%). According to a Swedish study (39) there is a declining gap between genders in committed crimes. The authors argue that there are multiple possible explanations to this, yet with the definitive consensus that there is an increase in females committing crimes. The present study was conducted at a high-security forensic psychiatric clinic with special admission criteria. The results might have been different if the study had recruited from lower-security clinical settings.

The mean age of onset within the different crime categories follow previous findings of criminal development in different forensic populations such as violent offenders (40), with the youngest mean ages of onset in drug related crimes (15.9 years) and theft or robbery (16.4 years), and the oldest mean age of onset for lethal violence (27.6 years). The age of onset for some of these crimes was in some cases as low as 5 years of age. Taken together, the criminal background of the participants seems characterized by a focus on violent criminality, yet with a versatility that must be seen in light of the context for recruitment: a high-security forensic psychiatric clinic. Thus, this pattern cannot be expected to translate to all forensic psychiatric contexts but may be specific to those referred to care facilities with high security. However, the results clearly demonstrate the need for early childhood interventions and support to prevent the criminological path of some forensic psychiatric patients, and for continued explorations of gender differences in criminological characteristics.

Representation of gender in the sample (predominantly male) was in line with reports from the Swedish National Registry of Forensic Psychiatry (16). The most common primary psychiatric diagnosis at the time of participation was schizophrenia spectrum and other psychotic disorder. While schizophrenia (paranoid and unspecified) was the overall most common (primary or secondary) diagnosis in this sample, over 64.3% of the participants had at some point during their lifetime been diagnosed with some kind of substance-related and addictive disorder. According to previous research, forensic psychiatric patients are more likely than a general psychiatric population to be treated with a combination of different antipsychotic medications and higher doses (19). For the current cohort of forensic psychiatric patients suffering from high comorbidity of psychotic disorders and substance use disorders, the pharmacological treatment could be immensely challenging for clinicians. Interestingly, the prevalence of neurodevelopmental disorders in this sample was lower at the time of participation than the lifetime prevalence. Although, symptoms of neurodevelopmental disorders might decrease with time for some individuals (41, 42), these disorders may not be adequately accounted for in forensic psychiatric care, since psychotic disorders, especially in a more acute phase, might overshadow other mental disorders. In fact, this could be a valid concern for many other mental disorders, such as personality disorders, as symptoms might be harder to tease out in an overall complex clinical picture. Regarding possible gender differences in psychiatric morbidity, it is known that women overall tend to report higher lifetime prevalence of mood and anxiety disorders (43) and BPD (44), and women in forensic psychiatry tend to be overrepresented in BPD (45). Although, we found that BPD was common in female participants, no such conclusions could be drawn from the current sample because of the low number of women represented. However, the results indicate differing psychiatric (co-)morbidity between female and male forensic psychiatric patients, something that needs to be explored in samples with a larger proportion of female patients before conclusions can be drawn. The current findings of a complex psychiatric comorbidity in forensic psychiatric patients emphasize the need for a forensic psychiatric care that accounts for both comorbidity and gender differences and tailor interventions accordingly.

### Prevalence, Characteristics, and Function of Self-Harm in Forensic Psychiatric Patients

The prevalence of self-harm in the current study was high, in line with previous studies on forensic samples (46, 47). More than half of the participants reported self-harm, including suicide attempts, at least once. This is a serious behavior that can lead to death or other serious physical injuries, and the consequences of self-harm are visible not only within health care or the individuals' personal suffering, but also in health economics. The societal costs of self-harm are often explained in terms of the costs, the need, and the length of hospitalization and/or medical treatment and psychosocial assessment related to the self-harm event (48). This study found three particularly interesting characteristics of self-harm: (1) hanging was the most common method of suicide attempt, (2) the most serious suicide attempt usually had no or minimal physical consequences, and (3) the most frequent form of NSSI was banging one's head or fist against a wall or cutting oneself.

Hanging as the most commonly used method of suicide attempt corresponds well with findings that hanging is the most frequently used method for completed suicide among men in Europe (49). Researchers argue that the chosen method of suicide is often influenced by the possibility of succeeding with the suicide without being detected (49). Results in our study show that most participants did choose a lethal method for their most serious suicide attempt, but they survived with minimal or no physical consequences. We suggest this might be because the suicide attempt was made in a forensic or care setting where the person had no possibility of being alone without supervision for any significant length of time. The suicide attempt, therefore, may not have been made with lethal intent, but could have had another function. However, since no detailed data on the circumstances around the suicide attempt were collected, this needs to be further investigated in future studies. Our findings can be contrasted against findings from patients with severe depressive disorders, where 32% had made a previous suicide attempt (50). We collected no further information on the circumstances or context of the most frequently used method of NSSI (banging fists or head against wall or cutting), but participants often told the data collector that these episodes of NSSI and/or suicide attempt had occurred during their arrest or early in their admission to forensic psychiatry. This provides increased support for the proposition that mentally disordered offenders are especially vulnerable to self-harm in critical time of their initial deprivation of liberty due to criminal offending and staff must be extra vigilant about the risk for self-harm in such contexts. All female participants reported some form of self-harm (NSSI and/or suicide attempt) and reported high frequencies of the NSSI behavior cutting, while male participants more frequently reported hanging/strangulation. Early studies argue for gender differences in self-harm behavior (51–53), while more recent studies [e.g., (54)] show that self-harm rates in men are not significantly different from those among women. Although, the current study showed a statistically significant difference between male and female participants concerning self-harm, general conclusions regarding gender differences cannot be drawn from this study because of the low number of female participants.

Previous studies on the functions of NSSI tend to fall on two sides: intrapersonal or interpersonal functions. The results of this study point to an intrapersonal orientation of the functions of NSSI, most prominently affect regulation, self-punishment, and distress signaling. This was especially prominent among female participants, although, the women also reported more interpersonal functions regarding interpersonal influence and self-care. This pattern is similar to that in discussions dominating the research field of self-harm today and shows that the functions of NSSI in forensic psychiatric patients, despite the influence of severe mental disorders, are comparable to those in other clinical and non-clinical groups (55–57) and that gender differences need to be considered. This information gives a unique insight into forensic psychiatric patients' perspectives on self-harm and is crucial for decisions on interventions directed toward self-harm in forensic psychiatry. Patients in forensic psychiatry also demonstrate, as evidenced earlier and in the current study, severe mental disorders and have also often experienced a traumatic childhood (58, 59).

### Psychosocial and Clinical Risk Factors of Self-Harm

There were no statistically significant associations between self-harm (NSSI and/or suicide attempts) and any of the psychosocial variables studied. Furthermore, no strong associations to any specific psychiatric diagnosis were demonstrated. However, self-harm was associated with neurodevelopmental disorders (*p* = 0.014, CI = 1.23–8.02, OR = 3.14) and disruptive impulse-control and conduct disorder (*p* = 0.012, CI = 1.19–74.6, OR = 9.41), although, the wide confidence intervals should be acknowledged. In numerous previous studies, self-harm has been associated with BPD, although, participants in the majority of those clinical studies have been female, and women are known to be overrepresented in BPD (44, 60, 61). In this study, we could not test the association between self-harm and BPD due to a low prevalence of the specific diagnosis in the sample. However, a high rate of self-harm was reported in several other diagnostic groups. Of the 67 participants who reported self-harm, 45 demonstrated a disorder within the spectrum of schizophrenia and other psychotic disorders. Even though, female gender increased the risk of self-harm 1.2 times, no gender-specific differences were demonstrated when the males in this sample were analyzed separately.

Given the NSSI functions reported by the participants, this could suggest they considered NSSI a way of expressing distress and frustration. However, conclusions about the function of NSSI must be drawn with caution and need to be further investigated in this particular group. Self-harm is a well-researched area, but not in forensic populations, and the differences in both the environmental and psychosocial backgrounds between a general population sample and a forensic sample must be taken into account.

### Strengths and Limitations

The sample in the current study was large considering previously reported difficulties in recruiting participants from forensic psychiatry (62), and the number of total forensic psychiatric patients existing in Sweden, representing ~5% of the total population and characteristics in line with the total population. However, the distribution of psychiatric diagnoses was not varied enough for analyses with self-harm as a dependent variable. Thus, in-depth analyses on self-harm in relation to possible risk factors were not feasible. Also, the current sample was recruited from a high-security forensic psychiatric clinic and may thus not be generalizable to forensic psychiatric settings in general. Differences in the legal context also need to be considered, since forensic psychiatric patients might be legally defined differently in other jurisdictions. Furthermore, we acknowledge the limitations due to sample size, affecting the statistical analysis possibilities. Also, since the current study was cross-sectional, no conclusions on causality can be drawn from the current findings.

Another limitation of this study is that the instrument used to collect self-report information on NSSI has not previously been used in a forensic sample. Although, the psychometrics of the instrument had acceptable values, this should be studied further. In the first part of the ISAS participants report the number of NSSI incidents. This becomes problematic in terms of reliability as the number rises as it did in our sample. Multiple participants reported more than 100 up to 1000 NSSI incidents. Without questioning the accuracy of their information, this result raises concern about whether this instrument is suitable for a sample with substantial NSSI. This has been pointed out as problematic in previous research (63, 64). Finally, we made no corrections for multiple comparisons, due to the study's explorative design.

### Conclusions

This study confirms forensic psychiatric patients as a vulnerable patient group with a complex and severe clinical presentation in combination with early maladjustment to society, where gender differences need to be considered. The results demonstrate that self-harm is a common and serious issue in a forensic psychiatric sample, with a higher prevalence than in the general population. Although, self-harm was significantly associated with neurodevelopmental disorders and disruptive, impulse-control, and conduct disorders, the confidence intervals were large in both cases and therefore no conclusions can be drawn in relation to clinical diagnosis. Self-harm was not associated with any specific psychosocial characteristics, but the predominant functions of NSSI in forensic psychiatric patients—affect regulation, self-punishment, and distress signaling—indicate that this group of vulnerable and exposed individuals may express their distress in a self-destructive manner.

## Data Availability Statement

The raw data supporting the conclusions of this article will be made available by the authors, without undue reservation.

## Ethics Statement

The studies involving human participants were reviewed and approved by Research Ethics Committee at Linköping University, 2016/213-31 and 2017/252-32. The patients/participants provided their written informed consent to participate in this study.

## Author Contributions

NL and MW developed the study concept. AO, SW, and ÅW contributed to the study design. Data collection and data analysis was performed by NL. NL drafted the paper. MW, AO, SW, and ÅW provided critical revisions. All authors approved the final version of the paper for submission.

## Conflict of Interest

The authors declare that the research was conducted in the absence of any commercial or financial relationships that could be construed as a potential conflict of interest.

## Publisher's Note

All claims expressed in this article are solely those of the authors and do not necessarily represent those of their affiliated organizations, or those of the publisher, the editors and the reviewers. Any product that may be evaluated in this article, or claim that may be made by its manufacturer, is not guaranteed or endorsed by the publisher.
