# Supplementary material for: Clinical Characteristics and Self-Harm in Forensic Psychiatric Patients
Source: Front Psychiatry. 2021 Aug 2;12:698372. doi: 10.3389/fpsyt.2021.698372 (PMC8365140; doi:10.3389/fpsyt.2021.698372)
Supplement: Supplementary file 1 [file Table_1.docx]

## Appendix

## Table A1. Psychosocial background of forensic psychiatric patients, stratified by gender*

| **Background characteristic** | | | **Male *n* (%)** | **Female *n* (%)** |  |
| --- | --- | --- | --- | --- | --- |
| **Born in Sweden** | | | 60 (70.6) | 10 (77) |  |
| **Marital status** | | |  |  |  |
|  | | Single | 74 (87.1) | 10 (77) | |
|  | | In a partner relationship/married | 10 (11.8) | 3 (23.1) | |
| **Parent of a child** | | | 23 (27.1) | 4 (30.8) |  |
| **Schooling** | | |  |  |  |
|  | | Graduated from primary school | 38 (44.7) | 5 (38.5) |  |
|  | | Truancy | 64 (75.3) | 10 (77) |  |
|  | | Bullied others | 27 (31.8) | 2 (15.4) |  |
| **Work experience** | | |  |  |  |
|  | | Full-time employment for >1 year | 31 (36.5) | 2 (15.4) |  |
|  | | Part-time employment for >1 year | 18 (21.2) | 1 (7.7) |  |
| **Upbringing circumstances** | | |  |  |  |
|  | | Parent(s) absent during childhood | 36 (42.4) | 4 (30.8) |  |
|  |  | Institutionalization before age 18 | 31 (36.5) | 5 (38.5) | |
|  |  | Foster care placement | 23 (27.1) | 5 (38.5) | |

*Males *n* = 85; females *n* = 13.

**Table A2. Criminological characteristics of forensic psychiatric patients, stratified by gender***

| **Type of offense** | **Yes**  **single occasion  *n* (%)** | | **Yes**  **repeated occasions  *n* (%)** | | **Age at onset *M* (range)** | |
| --- | --- | --- | --- | --- | --- | --- |
|  | **Male** | **Female** | **Male** | **Female** | **Male** | **Female** |
| Lethal violence | 16 (18.8) | 4 (30.8) | 5 (5.1) | 0 | 26.5 (19-39) | 33.8 (29-41) |
| Assaults (non-sexual) | 19 (22.4) | 3 (23.1) | 51 (60) | 8 (61.5) | 19 (5-47) | 21.9 (14-31) |
| Other violent crimes (non-sexual) | 12 (14.1) | 1 (7.7) | 66 (77.6) | 10 (76.9) | 22.7 (7-50) | 26.4 (14-41) |
| Sexual offences | 5 (5.9) | 1 (7.7) | 6 (7.1) | 0 | 23.5 (13-39) | 11 (-) |
| Theft or robbery | 17 (20) | 2 (15.4) | 62 (72.9) | 8 (61.5) | 16.3 (5-45) | 17.8 (8-45) |
| Economic offences | 10 (11.8) | 1 (7.7) | 14 (16.5) | 1 (7.7) | 22 (13-36) | 24 (20-28) |
| Traffic offenses | 26 (30.6) | 1 (7.7) | 38 (44.7) | 2 (15.4) | 20 (11-35) | 25 (16-35) |
| Drug offenses | 2 (2.4) | 2 (15.4) | 68 (80) | 8 (61.5) | 15.8 (8-35) | 17 (12-29) |
| Unlawful weapons possession | 21 (24.7) | 1 (7.7) | 34 (40) | 3 (23.1) | 20.6 (9-46) | 27 (13-47) |

*Males *n* = 85; females *n* = 13.

**Table A3. Current and historical mental disorders in forensic psychiatric patients, stratified by gender***

| **Diagnosis** | | **Lifetime prevalent diagnosis**  ***n* (%)** | | **Current primary diagnosis**  ***n* (%)** | | **Current secondary diagnosis**  ***n* (%)** | |
| --- | --- | --- | --- | --- | --- | --- | --- |
|  | | Male | Female | Male | Female | Male | Female |
| **Neurodevelopmental disorders** | | 38 (44.7) | 8 (61.5) | 18 (21.2) | 3 (23.1) | 18 (21.2) | 5 (38.5) |
|  | Intellectual disability, any kind | 10 (11.8) | 3 (23.1) | 3 (3.5) | 0 | 2 (2.3) | 2 (15.4) |
|  | Attention-deficit/hyperactivity disorder | 29 (34.1) | 5 (38.5) | 4 (4.7) | 0 | 13 (15.3) | 3 (23.1) |
|  | Autism spectrum disorder | 22 (25.9) | 3 (23.1) | 11 (13) | 3 (23.1) | 7 (8.2) | 0 |
| **Schizophrenia spectrum and other psychotic disorders** | | 64 (75.3) | 6 (46.1) | 46 (54.1) | 5 (38.5) | 7 (8.2) | 0 |
| **Bipolar and related disorders** | | 8 (9.4) | 3 (23.1) | 4 (4.7) | 1 (7.7) | 2 (2.3) | 0 |
| **Depressive disorders** | | 17 (20) | 7 (53.8) | 1 (1.2) | 0 | 0 | 0 |
| **Anxiety disorders** | | 20 (23.5) | 8 (61.5) | 0 | 0 | 0 | 0 |
| **Obsessive-compulsive and related disorders** | | 5 (5.9) | 2 (15.4) | 0 | 0 | 1 (1.2) | 0 |
| **Trauma- and stressor-related disorders** | | 12 (14.1) | 6 (46.1) | 2 (2.3) | 1 (7.7) | 0 | 0 |
|  | Post-traumatic stress disorder | 3 (3.5) | 5 (38.5) | 0 | 0 | 1 (1.2) | 1 (7.7) |
|  | Other trauma and stressor-related disorders | 10 (11.8) | 3 (23.1) | 0 | 0 | 2 (2.3) | 0 |
| **Disruptive, impulse-control, and conduct disorders** | | 13 (15.3) | 4 (30.8) | 1 (1.2) | 0 | 4 (4.7) | 1 (7.7) |
|  | Oppositional defiant disorder | 5 (5.9) | 0 | 0 | 0 | 1 (1.2) | 1(7.7) |
|  | Intermittent explosive disorder | 4 (4.7) | 1 (7.7) | 1 (1.2) | 0 | 0 | 0 |
|  | Conduct disorder | 4 (4.7) | 1 (7.7) | 0 | 0 | 0 | 0 |
|  | Unspecified disruptive, impulse-control, and conduct disorder | 6 (7) | 1 (7.7) | 0 | 0 | 2 (2.3) | 1 (7.7) |
| **Substance-related and addictive disorders** | | 55 (64.7) | 8 (61.5) | 2 (2.3) | 0 | 29 (34.1) | 3 (23.1) |
| **Personality disorders, any** | | 31 (36.5) | 11 (84.6) | 16 (1 9) | 2 (15.4) | 9 (10.6) | 3 (23.1) |
|  | Cluster A personality disorders | 6 (7) | 1 (7.7) | 0 | 0 | 0 | 0 |
|  | Cluster B personality disorders | 27 (31.8) | 11 (84.6) | 9 (10.6) | 3 (23.1) | 16 (19) | 2 (2.3) |
|  | Cluster C personality disorders | 0 | 1 (7.7) | 0 | 0 | 0 | 0 |
|  | Other personality disorders | 19 (22.3) | 6 (46.1) | 3 (3.5) | 1 (7.7) | 5 (5.9) | 0 |
| **Paraphilic disorders** | | 2 (2.3) | 0 | 1 (1.2) | 0 | 1 (1.2) | 0 |
| **Other mental disorders** | | 7 (8.2) | 3 (23.1) | 2 (2.3) | 0 | 0 | 0 |

*Males *n* = 85; females *n* = 13.

**Table A4. F****unctions of NSSI (mean ISAS values) in forensic psychiatric patients, stratified by gender**

| **ISAS scale** | **Function** | **Males** | | **Females** | |
| --- | --- | --- | --- | --- | --- |
|  |  | ***M (SD)*** | **Range** | ***M (SD)*** | **Range** |
| **Intrapersonal** |  |  |  |  |  |
|  | Affect regulation | 2.7 (2) | 0-6 | 4 (1.7) | 1-6 |
|  | Anti-dissociation | 1.3 (1.6) | 0-6 | 2.3 (2.2) | 0-5 |
|  | Anti-suicide | 1.2 (1.9) | 0-6 | 2.3 (2.3) | 0-6 |
|  | Marking distress | 1.8 (1.7) | 0-6 | 3.25 (2) | 0-6 |
|  | Self-punishment | 2.1 (1.7) | 0-6 | 3.25 (2) | 0-6 |
| **Interpersonal** |  |  |  |  |  |
|  | Autonomy | 0.5 (1.1) | 0-5 | 0.25 (0.9) | 0-3 |
|  | Interpersonal boundaries | 0.8 (1.3) | 0-4 | 1.1 (1.6) | 0-4 |
|  | Interpersonal influence | 1.2 (1.3) | 0-4 | 2.2 (1.9) | 0-5 |
|  | Peer bonding | 0.2 (0.5) | 0-2 | 0.2 (0.6) | 0-2 |
|  | Revenge | 0.3 (0.7) | 0-2 | 0.7 (1.3) | 0-4 |
|  | Self-care | 1.5 (1.8) | 0-6 | 3 (2.5) | 0-6 |
|  | Sensation seeking | 0.6 (1.3) | 0-6 | 0.6 (1.1) | 0-3 |
|  | Toughness | 1 (1.2) | 0-3 | 0.8 (1.3) | 0-3 |

*Males *n* = 85; females *n* = 13.

**Table A5. Psychosocial and clinical risk factors of self-harm (NSSI & suicide attempt) in male forensic psychiatric patients (*n* = 85)**

| Psychosocial and clinical characteristics | Self-Harm (*n*) | | | *ꭓ^2^* | *p* | CI | OR |
| --- | --- | --- | --- | --- | --- | --- | --- |
|  | **No** | **Yes** | **Expected yes-count** |  |  |  |  |
| Neurodevelopmental disorders | 8 | 30 | 24.1 | 7.05 | .008 | 1.36–9.45 | 3.60 |
| Schizophrenia spectrum and other psychotic disorders | 24 | 40 | 40.7 | 0.12 | .731 | 0.30–2.35 | 0.83 |
| Depressive disorders | 5 | 12 | 10.8 | 0.46 | .499 | 0.47–4.70 | 1.48 |
| Anxiety disorders | 5 | 15 | 12.7 | 1.49 | .223 | 0.65–6.20 | 2.00 |
| Trauma- and stressor-related disorders | 3 | 9 | 7.6 | 0.80 | .373 | 0.47–7.50 | 1.86 |
| Disruptive, impulse-control, and conduct disorders | 1 | 12 | 8.3 | 5.48 | .019 | 1.05–69.51 | 8.57 |
| Substance-related and addictive disorders | 21 | 34 | 34.9 | 0.19 | .657 | 0.32–2.06 | 0.81 |
| Personality disorder clusters A, B, and C | 13 | 18 | 19.7 | 0.63 | .428 | 0.28–1.72 | 0.70 |
| Cluster B personality disorders | 11 | 16 | 17.2 | 0.31 | .577 | 0.30–1.95 | 0.76 |
| Other personality disorders | 8 | 11 | 12.1 | 0.33 | .563 | 0.26–2.08 | 0.74 |
| Parents absent during childhood | 11 | 25 | 22.9 | 0.94 | .332 | 0.63–3.90 | 1.56 |
| Institutionalization during adolescence | 6 | 25 | 19.7 | 6.17 | .013 | 1.30–10.15 | 3.60 |
| Foster care placement during childhood | 6 | 17 | 14.6 | 1.46 | .226 | 0.66–5.52 | 1.91 |
| Truancy | 21 | 43 | 40.7 | 1.49 | .221 | 0.69–5.07 | 1.86 |
| Bullying others | 11 | 16 | 17.2 | 0.31 | .577 | 0.30–1.96 | 0.76 |
